# Identification of Methamphetamine Abusers Can Be Supported by EEG-Based Wavelet Transform and BiLSTM Networks

**DOI:** 10.1007/s10548-024-01062-2

**Published:** 2024-07-02

**Authors:** Hui Zhou, Jiaqi Zhang, Junfeng Gao, Xuanwei Zeng, Xiangde Min, Huimiao Zhan, Hua Zheng, Huaifei Hu, Yong Yang, Shuguang Wei

**Affiliations:** 1 Key Laboratory of Cognitive Science of State Ethnic Affairs Commission, College of Biomedical Engineering, South-Central Minzu University, Minzu Road, Wuhan, 430070 China; 2 Hubei Key Laboratory of Medical Information Analysis & Tumor Diagnosis and Treatment, Minzu Road, Wuhan, 430070 China; 3 Department of Radiology, Tongji Hospital, Tongji Medical College, Huazhong University of Science and Technology, Wuhan, 430030 China; 4 Department of anesthesiology, Tongji Hospital, Tongji Medical College, Huazhong University of Science and Technology, Wuhan, 430030 China; 5 School of Computer Science and Technology, Tiangong University, Tianjin, 300387 China; 6 Department of Psychology, College of Education, Hebei Normal University, Shijiazhuang, 050054 China

**Keywords:** Methamphetamine, Electroencephalogram, P300, Wavelet transform, Bidirectional long short-term memory

## Abstract

Methamphetamine (MA) is a neurological drug, which is harmful to the overall brain cognitive function when abused. Based on this property of MA, people can be divided into those with MA abuse and healthy people. However, few studies to date have investigated automatic detection of MA abusers based on the neural activity. For this reason, the purpose of this research was to investigate the difference in the neural activity between MA abusers and healthy persons and accordingly discriminate MA abusers. First, we performed event-related potential (ERP) analysis to determine the time range of P300. Then, the wavelet coefficients of the P300 component were extracted as the main features, along with the time and frequency domain features within the selected P300 range to classify. To optimize the feature set, F_score was used to remove features below the average score. Finally, a Bidirectional Long Short-term Memory (BiLSTM) network was performed for classification. The experimental result showed that the detection accuracy of BiLSTM could reach 83.85%. In conclusion, the P300 component of EEG signals of MA abusers is different from that in normal persons. Based on this difference, this study proposes a novel way for the prevention and diagnosis of MA abuse.

## Introduction

Methamphetamine (MA) is a neurological drug, which can cause serious mental symptoms, such as hallucinations and delusions. Light MA addiction can cause anxiety and other emotions, while severe MA addiction can cause depression and suicidal tendency (Zweben et al. 2004). Besides, some MA abusers suffer from psychosis and schizophrenia (Liu et al. 2017). Unfortunately, even though the drug is known to be too harmful to humans, little can be done about its addiction (Mooney et al. 2014). More unfortunately, unlike heroin and cocaine, MA formulas are available via the Internet and produced using common daily necessities (Lineberry and Bostwick 2006), which has accelerated its spread.

Electroencephalography (EEG) signals reflect the electrophysiological activity of nerve cells in the brain. In clinical practice, EEG signals have been shown to include a large number of physiological and pathological information (Zhong 2002). Such information can often give more details on a person?s physical condition, which has profound significance for both the prevention and remedy of diseases. In 1932, Dietch first used Fourier transform to analyze the collected EEG signals (Dietsch 2002). Since then, many analytical methods have been applied to study EEG signals, including time-domain and frequency-domain analysis, wavelet transform, artificial neural network (ANN) analysis and nonlinear dynamics analysis (Yousefi et al. 2022; Rafik and Ilyes 2023).

MA primarily affects the central nervous system. Therefore, consuming MA would lead to abnormal EEG signals of drug addicts (Prabhat et al. 2022; Gege et al. 2023). In this study, we attempted to record the EEG signals of MA abusers and analyze the differences in these signals between MA abusers and normal persons, which would support the exploration of the principle of action and treatment methods of MA.

As an important indicator of EEG signals, P300 is related to selective attention, memory renewal, motivation, stimulation significance and the activation of inhibition process (Turnip et al. 2013). Up to now, many studies have intensively investigated the detection of MA abuse using P300 (Jinxiang et al. 2013; Zhong et al. 2020; xxx yyy; Huang et al. 2023). MA abusers were asked to perform the same task as normal subjects, and it was shown that the P300 component can be successfully used to differentiate MA abusers from healthy subjects (Haifeng et al. 2015; Shuguang et al. 2018). With the advancements in neuroscience, researchers have developed a variety of feature extraction approaches of EEG signals to predict human psychiatric disorders (Shahmohammadi et al. 2016; Ahmadlou et al. 2013). However, little work has been so far devoted to extract the EEG P300 features of MA abusers to set up an automatic classification system and detect MA abusers.

EEG signals are non-stationary random signals (Shin et al. 2015), thus, conventional Fourier transform can only be used to see which frequency domains are EEG signals composed of, while the corresponding time information of each frequency component cannot be obtained. This means that there will be multiple identical time-domain graphs corresponding to one frequency-domain graph only. Hence, it is necessary to mention the wavelet transform, which can be used to decompose signals of different resolutions (Zhang 2020). By scaling and translation of the basis function, the window becomes narrower at high frequencies and wider at the low frequency, so that accurate frequency and time information can be obtained. Due to the superiority of the wavelet in signal transformation, wavelet transform is known as the “digital microscope” (Yu-Sheng et al. 2013). In view of the advantage of the wavelet transform, it was used as the extractor of time-frequency features of P300 signals to obtain wavelet coefficients as the main features (Jin et al. 2019).

Classical machine learning algorithms are commonly used methods for neurophysiological signal analysis and pattern recognition. The hypothesis in this study is that P300 signals in cognitive processes can demonstrate significant differences between MA abusers and normal persons. By extracting P300 features, the machine learning algorithm is able to recognize MA abusers.

The main contributions of this work are listed as follows:

1. For the first time, we used the features of the P300 component of EEG signals to detect MA abusers. Compared with other methods, the P300 component shows more differences between healthy people and MA abusers, and it is simpler to extract its features.
2. MA addiction could be detected through a few stimulation experiments, and the proposed model improved the detection efficiency.
3. Compared with biochemical detection methods, the detection results of our model were more reliable.

The remaining of this article is organized as follows. Chapter 1 mainly includes a short introduction to the research overview and background. In chapter 2, we present related work of EEG-based MA classification. Chapter 3 describes the experimental methods and process of data analysis. Chapter 4 presents the final results and related discussion, and a conclusion is presented in chapter 5.

## Related Work

### EEG-Based Research of Methamphetamine

MA can lead to substance use disorder (SUD), directly affect the central nervous system and impose a gigantic burden for the human body. The influence of MA on the brain can be mapped through the brain activity. EEG can reflect the activation process of the brain under various stimulation. It can enlarge the tiny biological electricity to a curvilinear record graph, recording the neural activity in ms. Its excellent temporal resolution ensures more precise analysis of neural activity.

Many studies have extensively investigated the effects of methamphetamine using EEG signals (Di et al. 2021; Chen et al. 2022; Lin et al. 2022). Khajehpour and colleagues explored the topological construction of the functional connectivity network at resting-state using the graph analysis method. The result of their research showed the network indicators, such as characteristic path length, clustering coefficient and small-world index at delta and gamma frequency bands, was different between MA abusers and healthy people (Hassan et al. 2019). Then, they extracted the features by the weighted phase lag index to identify the MA abusers using support vector machine (SVM) (Khajehpour et al. 2019). Shafiee-Kandjani proposed a potential difference in the coherence between different brain regions, which may be responsible for neuronal function defects and disorders (Shafiee-Kandjani et al. 2020). These studies indicated differences between MA abusers and healthy people regarding the brain functional connectivity network or cognitive behavior. Therefore, it is feasible to identify the MA abusers through these difference.

The above-mentioned methods have some disadvantages. Although they enable us to find a lot of differences between MA abusers and healthy people by analyzing the connection between different brain regions or consistency at different frequency bands, the algorithm to achieve fast feature extraction is too complex. From an engineering perspective, it is important that the implementation of the algorithm is as simple as possible. In this work, we proposed a feature extractor that calculates the time-frequency domain features. It utilizes the P300 component of EEG signals and diminishes the complexity of the algorithm to perform the calculation as fast as possible.

### Feature Selection

The feature extractor and classifier are two indispensable and important components in the machine learning-based EEG classification. Consequently, the correct feature extraction method and suitable classifier are particularly crucial. The features of time- and frequency-domain are important indices to measure the property of signals. This usually includes the maximum value, minimum value, mean value, mean frequency and so on. In general, the quantization is more intuitive in the time domain, while it is more informative in the frequency domain. They are interrelated and mutually advantageous. In addition, since EEG is a non-stationary signal, the time-frequency methods are also used as signal feature quantizer. In the work of Madhavan, the effect of using time-frequency features of EEG signals in epilepsy as the input for classification was investigated, and the sensitivity and specificity values of more than 99% were achieved (Madhavan et al. 2020). Ding and Howells (Ding et al. 2020; Howells et al. 2018) found MA abusers to have higher power or frequency activity at the delta band of EEG. Therefore, the P300 component located in the delta band was used for feature extraction.

Our model contain three kinds of features: time-domain features, frequency-domain features and time-frequency domain features. Those features can embody more comprehensive differences of the signals. However, it is widely accepted that redundant features can affect the performance of classification and introduce time consumption. It is necessary to perform feature selection and choose the better feature set.

### Bidirectional Long Short-Term Memory

For deep learning classification methods, Long Short-term memory network (LSTM), as a temporal recurrent neural network (RNN) suitable for processing and predicting important events with relatively long intervals and delays in a time series (Chen et al. 2016). The property of the forget gate within LSTM can selectively drop the features with poor classifying effect and retain the features that lead to good performance. Bidirectional LSTM (BiLSTM) is consisted by forward LSTM and backward LSTM. This design allows the LSTM to better capture the connection between the preceding and following information.

BiLSTM has been used in a variety of studies based on EEG. In motor imagery recognition studies, BiLSTM was mainly used to extract the time-series features from raw EEG signals (Hou et al. 2020; Lian et al. 2021). Yang proposed a method about EEG emotion classification by using BiLSTM, obtaining an experimental accuracy of 84.21% (Yang et al. 2020). Cui proposed a improved BiLSTM Multi-Fusion Model for emotion recognition, achieving an average accuracy of 94%? for online dataset (Cui et al. 2022). Zhong proposed a method that can use BiLSTM to detect epileptic seizures and impose the ultrasound deep brain stimulation to suppress them (Zhong et al. 2021). Zuo used BiLSTM to detect whether drivers were distracted while driving their vehicles (Zuo et al. 2022). In our model, we used BiLSTM to identify the EEG features distinguishing MA abusers from normal people.

## Materials and Methods

### Participants

In this study, we included 18 females with MA use disorder (recruited at an addiction rehabilitation center in Hebei Province, China) and 22 female healthy controls (recruited through community advertising). The inclusion criteria of female MA abusers were as follows: (1) The urine tested positive for MA before their drug abstinence, and abstinence was confirmed by the addiction rehabilitation center; (2) Meeting the diagnostic criteria for a history of MA abuse, no indication of being in early or sustained recovery and no dependence on substances (e.g., cocaine, heroin, marijuana) other than cigarettes and alcohol. This was assessed with the Structured Clinical Interview for the Diagnostic and Statistical Manual, fifth edition (DSM-V) (Michael et al. 2015; 3) No diagnosis of mental illness, with no past or present symptoms of psychiatric disorders; absence of brain trauma; (4) No use of psychotropic drugs within two weeks after the registration for this study. The healthy control group had no history of mental illness or substance abuse. In addition, all subjects accomplished the Sensation Seeking Scale-Version V (SSS-V) (Zuckerman 2007) and the Barratt Impulsiveness Scale-Version 11 (BIS-11) (Vasconcelos et al. 2012). All subjects had normal vision. The study protocol was approved by the Institutional Review Board of the Institute of Psychology of the Chinese Academy of Science, and all subjects obtained remuneration and agreed to the experiment. The demographic information and results of psychological tests for all subjects are detailed in Table 1.

**Table 1:** Sample characteristics

	HC group (n = 22)	MA group (n =18)	F1F1F1p values
Age (years)	27.05 ± 4.75	25 ± 4.41	0.17
Education (years)	9.15 ± 0.67	8.82 ± 2.16	0.51
Drug experience (months)	–	23.42 ± 10.05	
Abstinence time (months)	–	14.53 ± 3.84	
Methamphetamine use, lifetime (g)	–	266.13 ± 407.42	
Number of cigarettes per day	–	8 ± 8.27	
Alcohol use per day (g)	–	23.03 ± 63.23	
BIS-11	63.75 ± 11.02	69.84 ± 9.83	0.08
Cognitive impulsiveness	18.3 ± 5.18	17.07 ± 2.99	0.37
Motor impulsiveness	20.56 ± 3.97	23.28 ± 3.78	$<0.05^{*}$
Non-planning impulsiveness	25.27 ± 5.55	29.49 ± 5.51	$< 0.05^{*}$
SSS-V	12.7 ± 4.07	17.32 ± 4.85	$$<0.01^{**}$$
Disinhibition	2.35 ± 2	4.16 ± 2.54	$$<0.01^{**}$$
Experience seeking	3.6 ± 1.9	5.23 ± 1.65	$$<0.01^{**}$$
Thrill and adventure seeking	4.5 ± 2.97	5.39 ± 2.19	0.3
Boredom susceptibility	2.25 ± 1.4	2.56 ± 1.4	0.5

*BIS-11* Barratt Impulsiveness Scale, Version 11; *SSS-V* Sensation Seeking Scale Form V

*p $$<0.05$$

**p $$<0.01$$

### Procedure

In this study, the pattern of our task was the modified two-choice oddball paradigm (Jiajin et al. 2009). The task contained three blocks, such that 70 standard basketball pictures is contained by each block, labeled S1, and 30 deviant pictures, consisting of 15 pictures of addiction (e.g., MA, MA paraphernalia and MA smoking) and 15 neutral pictures, labeled S2 and S3, respectively. The MA-related pictures and neutral ones were taken from social media, and they were matched for the social content.

All subjects were directed to choose the correct answers rapidly. They would show their quick response under the frequent presence of images when deviant stimuli were present. The subjects were notified this information before the experiment. The subjects were seated in a quiet compartment with the computer screen about 80 cm away from them. Then, each trial began with keeping the eyes fixed at the center of a small white cross for 300 ms on a black screen. Subsequently, the small white cross will disappear for a moment, the time of disappearing varied randomly from 500 to 1500 ms, followed by the beginning of the image stimulus. The subjects were asked to press the “F” (or “J”) key on the keyboard as accurately and quickly as possible if the standard picture appeared and the “J” (or “F”) key if the deviant picture appeared (counterbalanced among participants). The subjects were told that their response must be less than 1000 ms, since the stimulus would be terminated by a keystroke or by itself. There was a 1000 ms black screen for break after each response.

**Fig. 1:**
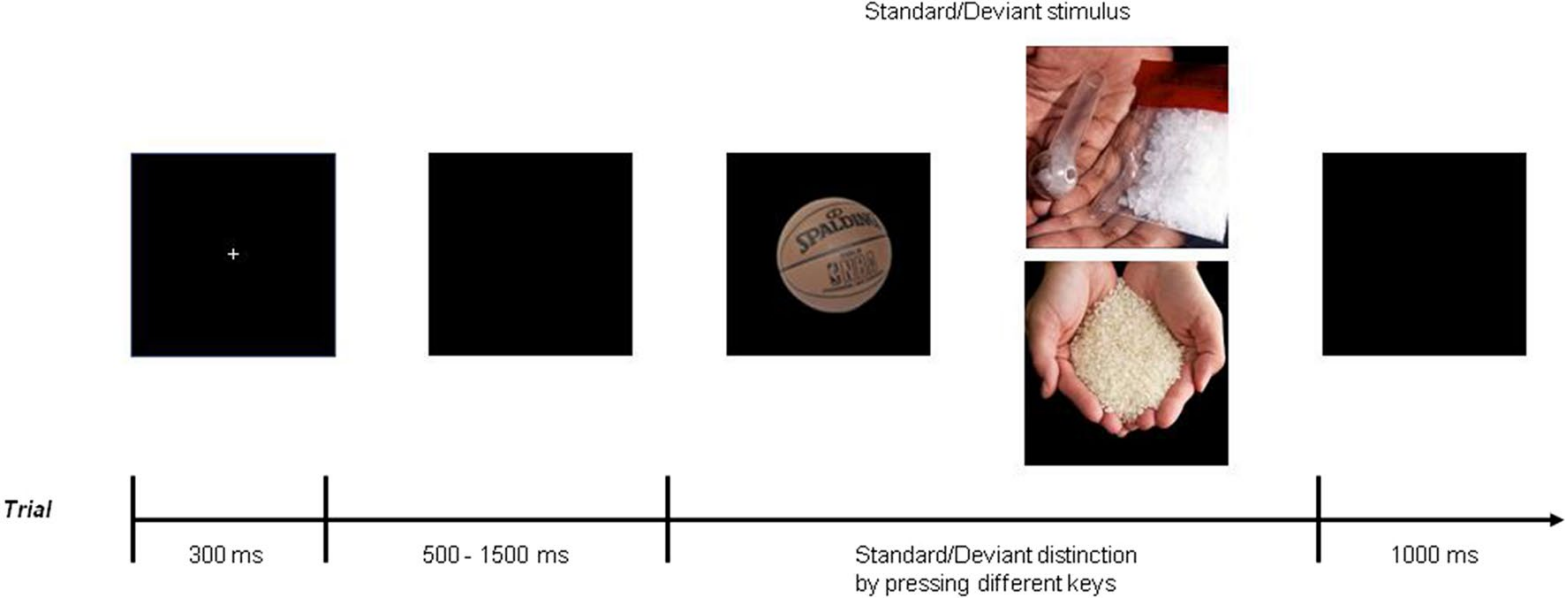
Schematic illustration of the behavioral procedure and stimulus examples in a trial

Before the formal experiment, procedure will provide 15 practice experiments for subjects and they were asked to familiarize the process. The standard picture was identical under two pattern (training and formal), while the deviation picture of the training was different from those selected in the formal trial. Both of experiments were necessary to each subject, all subjects completed perfectly the practice trials before starting with the formal trials. The experiment flow is shown in Fig. 1.

### Data Recording and Preprocessing

Continuous EEG was recorded using the Brain Vision Recorder 2.0 system (Brain Products Company, Munich, Germany). In the process of recording, FCz was regarded as the reference electrode, and the ground electrode was AFz. An electrode placed approximately 2 cm below the right eye and centered under the pupil was used to record vertical electrooculogram (EOG). The recorded EEG signals were amplified and digitized at a sampling rate of 1000 Hz in the DC acquisition mode, and the electrode impedance was required below 10 k$\Omega$.

**Fig. 2:**
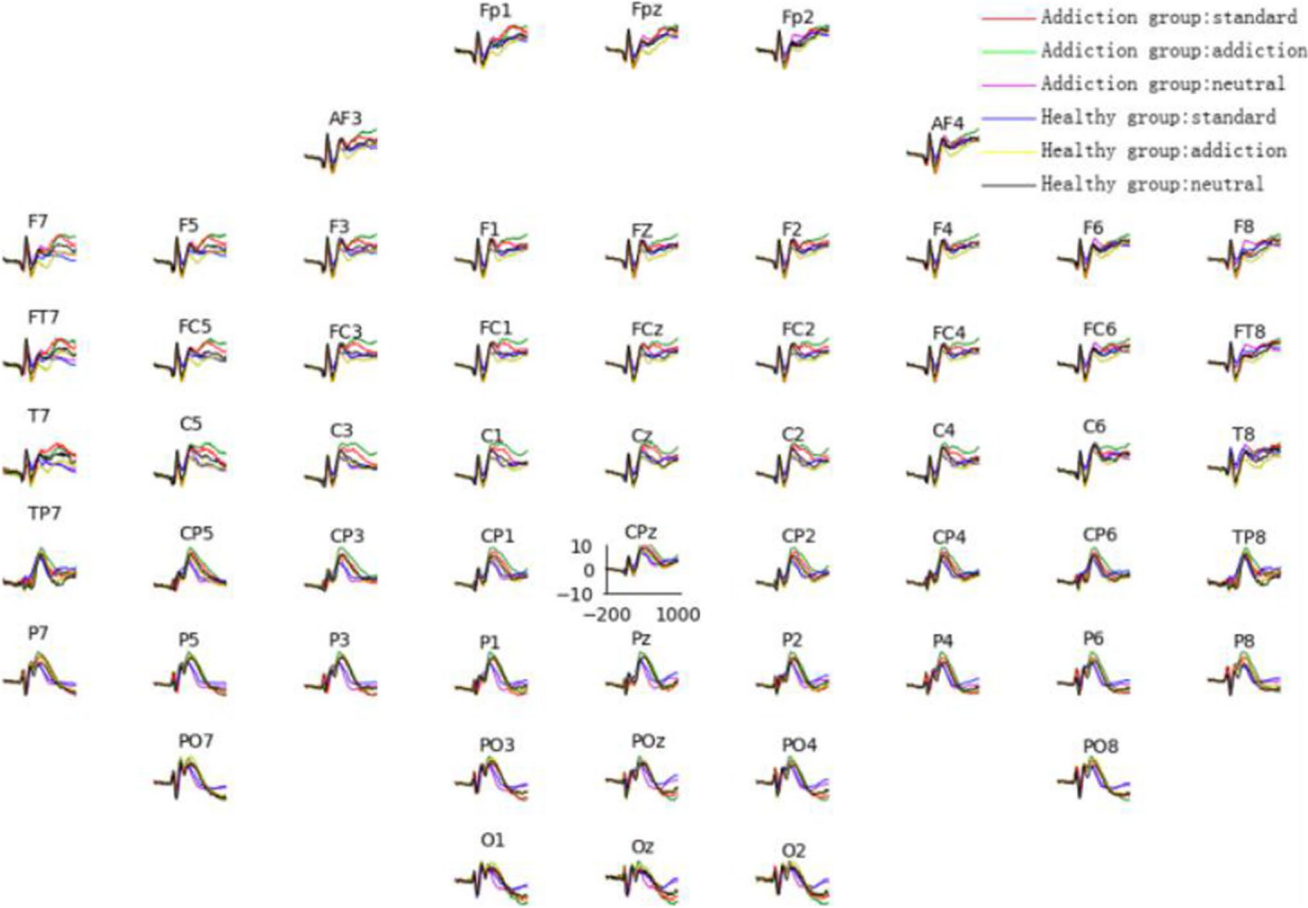
ERP waveform of each channel under addiction stimulation

The data were processed offline after recording. The operation of processing was executed by EEGLAB and ERPLAB toolboxes based on the MATLAB Platform. Data were re-referenced to a mastoid electrode averaged reference, down_sampled to 250 Hz, filtered by a 30 Hz low-pass filter. Artifacts, such as including spikes, EEG drift and abiotic signals were manually removed. Then the eye electrical interference was eliminated through independent component analysis (ICA). The range of each epoch was $-200$ to 1000 ms, and we used 200 ms before stimulus presentation as baseline correction. The event-related potential (ERP) of each channel under three stimulations after preprocessing is shown in Fig. 2. As shown in Fig. 2, there was a big difference in the ERP between two groups, especially in the P300 under S2 stimulation. Therefore, only the epochs of S2 were extracted, and every five epochs of each subject were overlapped and averaged. Finally, 144 groups of data were obtained in the addiction and healthy groups, respectively.

### Discrete Wavelet Transforms (DWT) for Signal Analysis

Most biological signals in nature are non-stationary random signals; hence, wavelet transform is usually used in biomedical signal field. The time domain features, frequency domain features and wavelet coefficients of P300 stimulated by the S2?signal were abstracted for classification in this work.

The wavelet transform is operated by two basic functions: scale function $\phi (t)$ and wavelet function $\psi (t)$, which are the prototype forms of the following class of orthonormal basis functions, respectively:

1 $$\begin{aligned}{} & {} {\phi _{j,k}}(t) = {2^{j/2}}\phi ({2^j}t - k);j,k \in Z \end{aligned}$$

2 $$\begin{aligned}{} & {} {\psi _{j,k}}(t) = {2^{j/2}}\psi ({2^j}t - k);j,k \in Z \end{aligned}$$

where *k* controls the translation of the wavelet base in the time domain, *j* denotes the parameter in the frequency domain, which determines the frequency features of the wavelet base, and *Z* is a set of integers.

The complete wavelet expansion *f*(*t*) is defined by the wavelet function and the scale function, as follows:

3 $$\begin{aligned} f(t) = \sum \limits _{{j_0} \in Z}^\infty {c({j_0},k)} {\phi _{{j_0},k}}(t) + \sum \limits _{j > {j_0}} {\sum \limits _{k = 0} {d(j,k)} } {\psi _{j,k}}(t) \end{aligned}$$

where the coefficients $c(j_0,k)$ and *d*(*j*, *k*) are calculated by inner product as follows:

4 $$\begin{aligned}{} & {} c({j_0},k) = \left\langle {f(t),{\phi _{{j_0},k}}(t)} \right\rangle \end{aligned}$$

5 $$\begin{aligned}{} & {} d(j,k) = \left\langle {f(t),{\psi _{j,k}}(t)} \right\rangle \end{aligned}$$

This is the final and core form of the wavelet transform (Gandh et al. 2010). In this study, the quadratic B-spline function (Gan et al. 2017) was selected as the mother wavelet. The N-order approximate coefficients can be calculated by wavelet decomposition. Given that the samples are 250 Hz, a fifth-order wavelet decomposition is performed. In the wavelet transform multi-resolution algorithm, low-pass (LP) and high-pass (HP) filters use the same wavelet coefficients. The coefficients with LP filter are related to the scaling function. Its outputs are called the approximate quantity (A). While the HP filter have a connection with the wavelet function, and its outputs are called detail quantity (D).

### Feature Extraction

The frequency range of P300 has been confirmed to be in the delta band (Gao et al. 2010). Therefore, using the method of wavelet decomposition, we calculated the wavelet coefficients of the delta band as the wavelet features. Furthermore, the time and frequency domain features of all signals *X*(*t*) have also previously been added to classify the two types of signals (Gao et al. 2014). Several time domain features were calculated as follows:

1. Maximum amplitude (MAA): the maximum amplitude of *X*(*t*), calculated as follows:
  6 $$\begin{aligned} MAA = \max \{ X(t)\} \end{aligned}$$
2. Minimum amplitude (MIA): the minimum amplitude of *X*(*t*), calculated as follows:
  7 $$\begin{aligned} MIA = \min \{ X(t)\} \end{aligned}$$
3. Latency (LAT): the time at which MAA of *X*(*t*) occurs, calculated as follows:
  8 $$\begin{aligned} LAT = \{ X(LAT) = MAA\} \end{aligned}$$
4. Ratio latency to maximum (RLM): the ratio latency to maximum amplitude of *X*(*t*), calculated as follows:
  9 $$\begin{aligned} RLM = \frac{{MAA}}{{LAT}} \end{aligned}$$
5. Positive area (PA): the sum of the positive signal values of *X*(*t*), calculated as follows:
  10 $$\begin{aligned} PA = \sum \limits _{t1}^{t2} {\frac{{X(t) + \left| {X(t)} \right| }}{2}} \end{aligned}$$
  where *t*1 and *t*2 denote the initial time value and cut-off time value of P300, respectively.
6. Difference between positive and negative amplitude (DPN), calculated as follows:
  11 $$\begin{aligned} DPN = MAA - MIA \end{aligned}$$

Let *Y*(*f*) be the power spectral density of *X*(*t*). Then, the following calculation methods can be used to extract the frequency domain features:

1. Maximum frequency (MF): the maximum frequency of *X*(*t*), calculated as follows:
  12 $$\begin{aligned} MF = \{ Y(MF) = Y{(f)_{\max }}\} \end{aligned}$$
2. Average frequency (AF): calculated by a frequency weighted average, where the weighted coefficient is the value of *Y*(*f*). It can be calculated as follows:
  13 $$\begin{aligned} AF = \frac{{\int _0^{125} {f \times Y(f)df} }}{{\int _0^{125} {Y(f)df} }} \end{aligned}$$

**Fig. 3:**
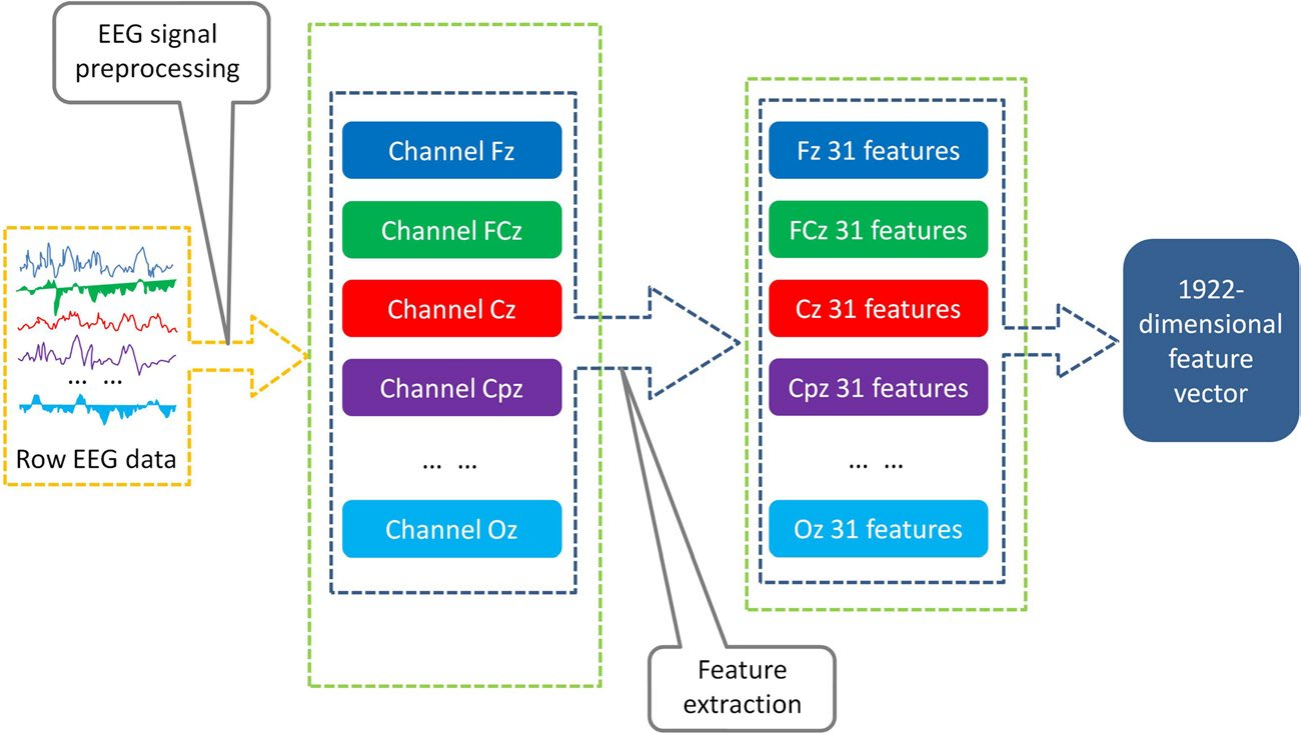
The block diagram for the entire processing pipeline of feature extraction

A total of 31 features under each channel were involved in the classification, and an EEG sample with features from 62 channels was a 1922-dimensional vector. The entire feature extraction mechanism is summarized in Fig. 3, and the pseudo-code of the algorithm with feature extractor is shown in Algorithm 1.

However, a lot of repetitive or redundant information exist in EEG signals (Amin et al. 2015), so there is a strong correlation between the features, which can weaken the generalization ability of the model and reduce the classification accuracy. Thus, the F_score is still needed to select which features would have better classification effect.

**Algorithm 1:**
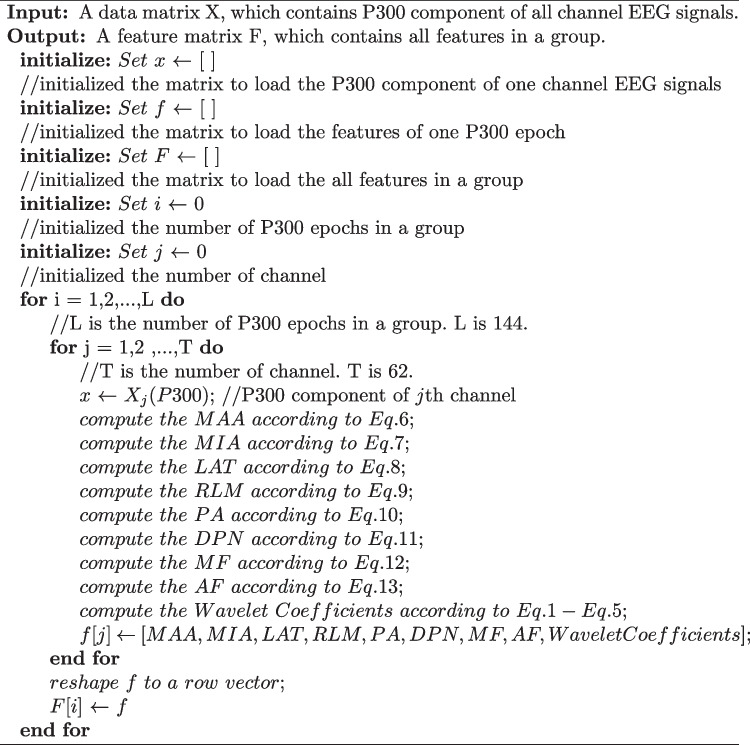
Design of the feature extractor

### F_score for Feature Selection

F_score can measure the discrimination of two sets of features (Xie et al. 2010). Let ${x_k} \in {R^m},k = 1,2,\ldots ,n$, be the given recording set, and the number of positive and negative samples be ${n_ + }$ and ${n_ -}$, respectively. Then, the F_score value of the *i*th feature of the training sample can be defined as follows:

14 $$\begin{aligned} F(i) = \frac{{{{(\overline{{x_i}^{( + )}} - \overline{{x_i}} )}^2} + {{(\overline{{x_i}^{( - )}} - \overline{{x_i}} )}^2}}}{{\frac{1}{{{n_ + } - 1}}\sum \limits _{k = 1}^{{n_ + }} {{{({x_{k,j}}^{( + )} - \overline{{x_i}^{( + )}} )}^2} + \frac{1}{{{n_ - } - 1}}\sum \limits _{k = 1}^{{n_ - }} {{{({x_{k,j}}^{( - )} - \overline{{x_i}^{( - )}} )}^2}} } }} \end{aligned}$$

where ${{x_{k,j}}^{( + )}}$ and ${{x_{k,j}}^{( - )}}$ are the *k*th positive and negative sample points of the *i*th feature, respectively. ${\overline{{x_i}} }$, ${\overline{{x_i}^{( + )}} }$ and ${\overline{{x_i}^{( - )}} }$ represent the average of the whole, positive and negative data sets, respectively. The degree of the discrimination of the feature is decided by the value of the F_score (Chen and Lin 2006).

In this study, F_score was calculated for all features, and we removed the features with a score below the average score. All features will be put into the feature set in descending order to form the final feature set. The feature selection algorithm is shown in Algorithm 2.

**Algorithm 2:**
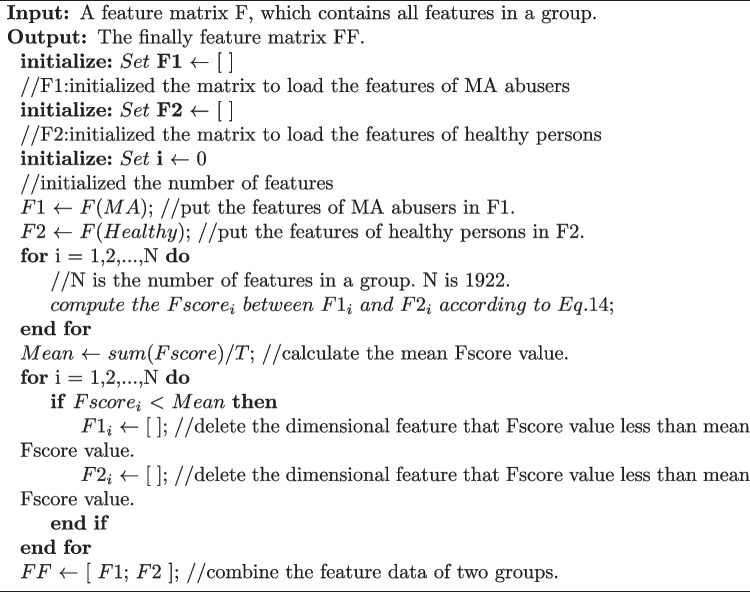
Feature selection

### Classification

**Fig. 4:**
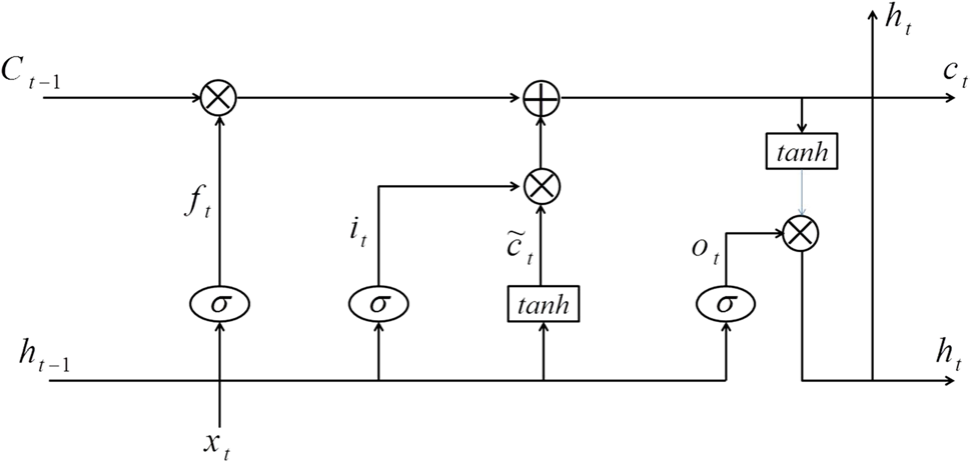
The memory unit structure of BiLSTM

LSTM has shown a good effect in a variety of applications in image recognition, text analysis, disease prediction and so on (Zhang et al. 2019). LSTM is an improved and upgraded RNN. It adds weight control over different moments of memory through a gate controller. The memory unit structure of LSTM is shown in Fig. 4.

*c* represents a memory state, which is the most important part of the entire memory unit (Houdt et al. 2020). As shown in Fig. 4, it is directly transferred on the entire structural chain, using only a small number of linear operations, hence the information is substantially unchanged during transmission. Meanwhile, the memory unit contains three intelligent “gate” structures to control the flow of information, namely forget gate input gate and output gate. According to the choice of the gate, the information contained in the memory status can be added or deleted. It includes a point-by-point multiplication operation of vectors and a sigmoid function, mainly with the following parts:

${C_{t - 1}}$ represents the memory state at time $t-1$, which records historical information of all time steps. It belongs to the long-term memory of the model.

${h_{t - 1}}$represents the output at time $t-1$, which mainly records the time step information, thus belonging to the short-term memory of the model.

Forget gate $f_t$, input gate $i_t$ and output gate $o_t$, are between 0 and 1. The memory state *C* of the *j*th memory unit at time *t* is the operated result of the input gate $i_t^j$, forget gate $f_t^j$ and previous memory state $C_{t-1}^j$ (Zaremba et al. 2014). The memory status *C* at time *t* is defined as:

15 $$\begin{aligned} C_t^j = f_t^j \times C_{t - 1}^j + i_t^j \times {\tilde{C}}_t^j \end{aligned}$$

such that

16 $$\begin{aligned} \left\{ \begin{array}{l} {f_t} = \sigma ({W_f}[{h_{t - 1}},{x_t}] + {b_f})\\ {i_t} = \sigma ({W_i}[{h_{t - 1}},{x_t}] + {b_i})\\ {{{\tilde{C}}}_t} = \tanh ({W_C}[{h_{t - 1}},{x_t}] + {b_C}) \end{array} \right. \end{aligned}$$

where *w*, *b*, $\sigma$ and $x_t$ represent the weight matrix, bias parameters, sigmoid activation functions corresponding to each gate, and input of the model at time *t*, respectively. When the memory unit is updated, the output gate $o_t$ and the hidden layer $h_t$ can be represented as:

17 $$\begin{aligned} \left\{ \begin{array}{l} {o_t} = \sigma ({W_O}[{h_{t - 1}},{x_t}] + {b_O})\\ {h_t} = {o_t} \times \tanh ({C_t}) \end{array} \right. \end{aligned}$$

**Fig. 5:**
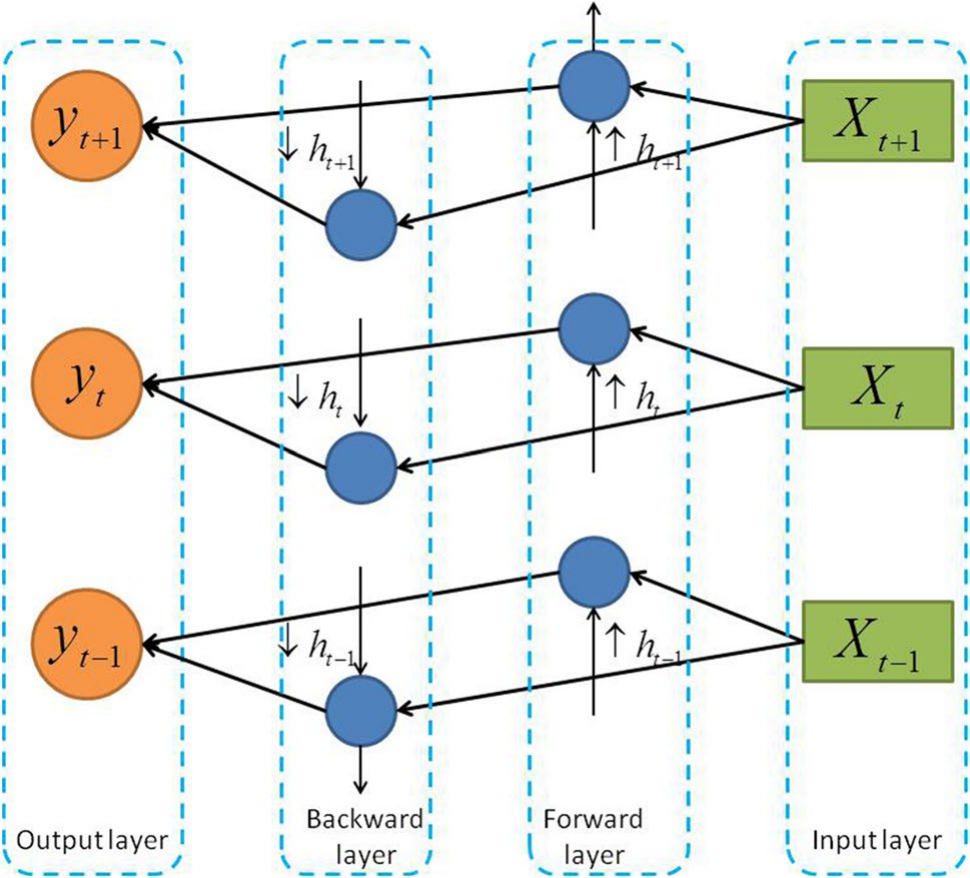
The architecture of the BiLSTM model

Traditional LSTM networks mainly use historical background information. However, lacking future feature information may lead to an incomplete feature matrix. As for BiLSTM, it realizes bi-directional features reading by combining the forward and backward LSTM layers, hence fully taking the context feature information (Zhang et al. 2020). The model structure is shown in Fig. 5.

Given that at time *t*, $\uparrow {h_t}$ is a hidden state of the forward LSTM output, and $\downarrow {h_t}$ is a hidden state of the backward LSTM, then $h_t$ can be calculated as follows:

18 $$\begin{aligned} {h_t} = \uparrow {h_t} \oplus \downarrow {h_t} \end{aligned}$$

Studies have proven that the BiLSTM model containing overall information is better than other classification algorithm (Wang et al. 2016).

### Cross-Validation

To ensure that all data can be used for classification, we performed 12-fold cross-validation (CV) (Xu and Goodacre 2018). First, we randomly divided all the feature samples into 12 sample sets. Within these 12 sets, 11 sets were used to train model, and can be denoted by ${D_{tra}}$, while the 12th set was selected to test model, and denoted by ${D_{tes}}$. We repeated this process 12 times, with each set being used once for testing. Furthermore, we applied an additional 8-fold cross-validation on each ${D_{tra}}$ set, randomly dividing each set ${D_{tra}}$ into 8 subsets, where 7 sets served as training subsets (${D_{s\_tra}}$) and the 8th set served as the validation subset (${D_{s\_val}}$). ${D_{s\_tra}}$ and ${D_{s\_val}}$ were then assigned to the classifier for training and validating its performance. This process was repeated 8 times, with each subset being used once for validation, to overcome the overfitting problem. We used the following performance measures for classification:

- Balanced validation accuracy (BVA): For each Dtra, BVA was obtained by averaging 8 pairs of sensitivities and specificities (8-fold CV). By comparing the classification result of different feature sets, the best BVA can be obtained and the optimal classifier (and its parameters) could be obtained as the one with the highest BVA. It is worth knowing that the best results maybe have some differences for a different set ${D_{tra}}$.
- Balanced Testing Accuracy (BTA): BTA was obtained by averaging the sensitivity and specificity, and the sensitivity and specificity is the test result of the classifier which have trained completely. Through the 12-fold CV, we could obtain the average of all 12 BTAs as ABTA (average BTA). Then, the final optimal classifier (and its parameters) was decided when the BTA reached the highest among all the 12 BTAs.

**Fig. 6:**
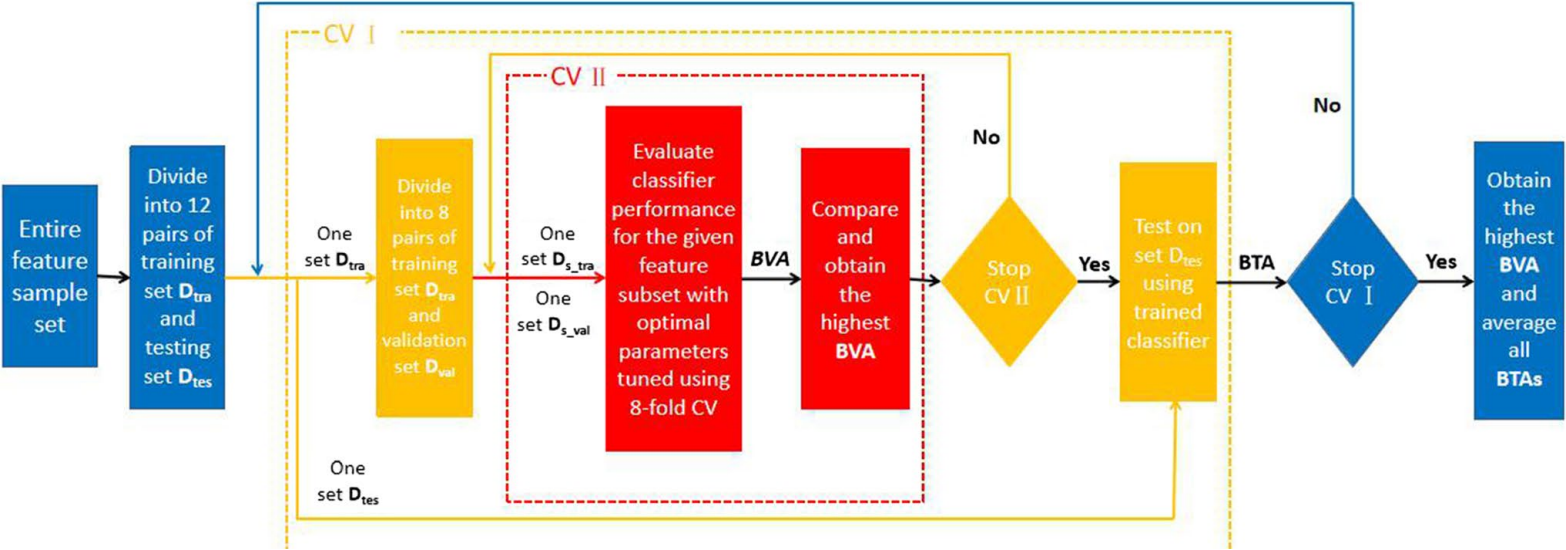
Flow chart of the classification. The operation results and data are expressed in italics. CV *i*: The first layer cross-validation, testing the optimal model of the second layer CV training. CV *II*: The second layer cross-validation, training the best model and verify. The two branches of orange lines represent the input of the training set and testing set in the first layer CV, respectively. The same is true for the meaning of the red line in the second layer CV

The cross-validation illustration is shown in Fig. 6. The feature sample set was formed after the F_score feature selection operation, and after two layers of CV, the best training model and the average test accuracy were obtained.

### Statistical Evaluation of Performance

For classification models, the test accuracy is a very good and intuitive evaluation indicator. However, accurate rates do not always fully represent a model as good or bad. Therefore, the performance of the classifier was also evaluated by the following specificity (Spe) and sensitivity (Sen) metrics (Subasi 2007):

19 $$\begin{aligned}{} & {} Sensitivity = \frac{{TP}}{{(TP + FN)}} \times 100\mathrm{{\% }} \end{aligned}$$

20 $$\begin{aligned}{} & {} Specificity = \frac{{TN}}{{(TN + FP)}} \times 100\mathrm{{\% }} \end{aligned}$$

where sensitivity is the true positive rate, reflecting the probability of missed diagnosis, and specificity is the true negative rate, reflecting the probability of misdiagnosis. The following statistics are used to calculate specificity and sensitivity:

TP (True Positive): the number of addiction cases identified by the classifier, which are actually addiction cases.

FN (False Negative): the number of healthy cases identified by the classifier, which are actually addiction cases.

TN (True Negative): the number of healthy cases identified by the classifier, which are actually healthy cases.

FP (False Positive): the number of addiction cases identified by the classifier, which are actually healthy cases.

## Results

### Classifier Performance

In this study, 288 cases were classified, including 144 in the addiction group and 144 in the healthy group.

**Fig. 7:**
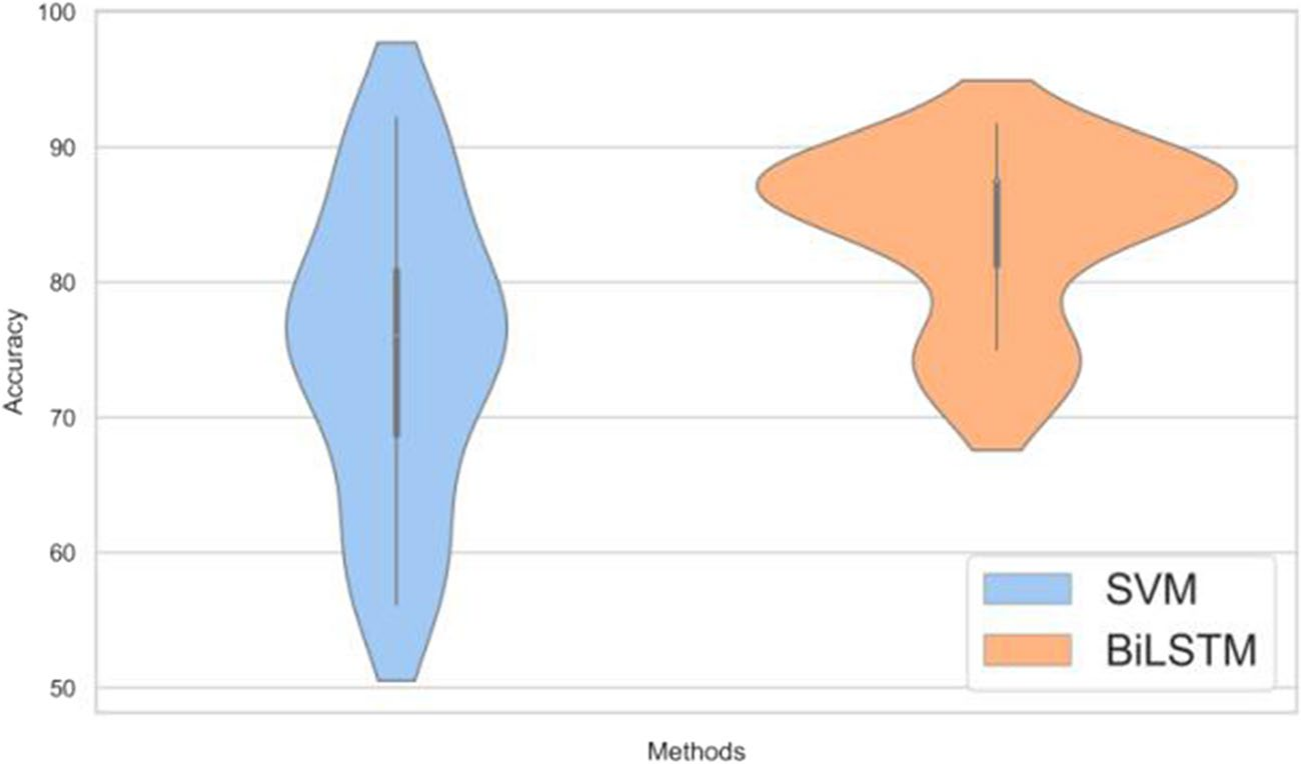
Violin plot of the test accuracy with the average accuracy of different classifiers

The time-domain features, frequency-domain features and wavelet coefficients of the delta band were used as classification features. Then, the features of 62 channels were connected in series. To improve the performance of classifier and reduce the computation and experiment time, F_score was used to evaluate the discrimination degree of the feature data. Next, arranging features in descending order, and classification was performed. In this study, the classification accuracy of SVM and BiLSTM were compared. To increase the comparability, all BiLSTM and SVM classifiers used the sigmoid activation function. The test accuracy of 12-fold cross-validation was calculated, and the violin diagram was plotted (see Fig. 7). It can be seen that the classification results obtained by BiLSTM in cross-validation are more stable compared with SVM.

Table 2 shows the performance evaluation results of the feature dimension with the highest classification accuracy after classifying the data with different classifiers. It was observed that when BiLSTM was used as the classifier, the classification accuracy was better. The P300 features of EEG signals were used to classify MA abusers and healthy subjects, and we achieved a classification accuracy rate of 83.85%.

**Table 2:** The evaluation indicator of the best models in different classifiers

Classifier	Average training accuracy (%)	Average testing accuracy (%)	Training error (%)	Testing error (%)	Sen (%)	Spe (%)
SVM	89.17 ± 1.25	75.0 ± 11.19	10.83 ± 1.25	25.0 ± 11.19	78.14 ± 15.23	71.86 ± 13.52
BiLSTM	94.47 ± 0.76	83.85 ± 6.16	5.53 ± 0.76	16.15 ± 6.16	81.15 ± 8.81	86.2 ± 8.85

### Performance of Different Electrodes

In addition, due to the characteristics of BiLSTM, we also compared the use of different electrode signals separately for classification. Among them, 20 electrodes achieved a classification accuracy of 60% (see Table 3). It can be seen from Table 3 that most of the electrodes were located in the frontal?lobe, to which the advanced functions of brain, such as judgment, decision making, thinking and executive control are related (Anne et al. 2012). Therefore, the signal of the frontal?lobe can more clearly reflect the mental activity of the two groups during the task implementation. The C3 and C4 electrodes were substantially mapped in the primary motor cortex region, and the region electrode signal also exhibited strong differences. This may indicate that MA has an impact on the function of exercise execution in MA abusers. Meanwhile, this phenomenon may also be related to the symptoms in patients with amphetamine psychosis, such as irritation, anxiety and psychomotor agitation.

**Table 3:** The classification results of different electrode signals using BiLSTM

Channel	accuracy (%)	Channel	Accuracy (%)	Channel	accuracy (%)	Channel	Accuracy (%)
Fp1	67.53 ± 5.53	FC5	63.89 ± 6.03	FC3	62.15 ± 5.57	C4	60.42 ± 6.51
F7	64.76 ± 6.34	AF3	63.72 ± 7.43	AF4	61.81 ± 7.31	F5	60.42 ± 8.21
C5	64.58 ± 5.32	FC1	63.19 ± 7.11	AF7	61.46 ± 7.63	FC2	60.42 ± 5.03
FCz	64.24 ± 6.47	Fpz	63.19 ± 5.62	PO8	61.28 ± 5.32	FT9	60.24 ± 6.73
F1	64.06 ± 7.01	Fz	62.85 ± 4.32	C6	60.94 ± 6.21	C3	60.07 ± 7.69

## Discussion

### Identification of MA Addiction Based on EEG

The previous research indicates that men and women exhibit similar MA-related characteristics and behaviors (Brecht et al. 2004). Compared with men, women^′^s symptoms were more specific to MA and they responded better to treatment (Dluzen and Liu 2008). Therefore, we collected EEG signals from 18 female perpetrators and 22 healthy females, processed using MATLAB R2014b software and the EEGLAB toolbox. After preprocessing, we obtained 144 and 184 groups of data, respectively. Figure 2 illustrates significant differences between the signals of the two groups. The ERP amplitudes corresponding to MA-related stimuli in MA addicts are higher than those in the control group. Studies have shown that MA addicts exhibit attentional bias towards MA-related cues when exposed to such stimuli (Gege et al. 2023; Di et al. 2021). Our results are consistent with previous studies.

Currently, common methods used in EEG-based MA addiction research include traditional resting-state EEG analysis, brain network analysis, and event-related potential analysis (Di et al. 2021; Chen et al. 2022; Lin et al. 2022; Hassan et al. 2019; Khajehpour et al. 2019; Shafiee-Kandjani et al. 2020; Li et al. 2022). A few studies classify MA addicts and healthy individuals based on features extracted from resting-state EEG or event-related potentials (Zolfaghari et al. 2024; Xiong et al. 2019). We classified EEG signals of MA addicts and healthy subjects for the first time based on 31 features. Using SVM as the classifier, we achieved a highest classification accuracy of 72.30% (Xiong et al. 2019). In this paper, we further selected more features and achieved 75% accuracy in SVM. Meanwhile, utilizing the BiLSTM classifier in deep learning, we achieved an accuracy of 83.85%.

During the classification process, since we needed to match the data of the two groups, we had to discard a portion of the healthy group data, resulting in each group having 144 elements. Increasing the data volume may potentially lead to improved classification performance.

In this experiment, we mainly focused on the time-domain, frequency-domain features and wavelet analysis of P300, and there was no in-depth study on other related components. Other indicators may have better classification features.

### Engineering Application

MA is a highly addictive drug, which can cause nervous confusion and hyperactivity. Due to its availability and low cost, it has become very popular among the crowd, and it is closely linked to high crime rates around the world. It is necessary to test for MA addiction because its withdrawal is very difficult. However, many MA abusers attempt to use negative strategies to influence the biochemical tests of their addiction. Therefore, adding other test items based on biochemical tests can enhance the reliability of test results. Since it is difficult to artificially control the neural activity of the brain through training, it can be very useful to optimize an EEG ERPs-based detection method for MA addiction. However, it should be emphasized that our method serves as a supplementary approach based on biochemical testing to enhance the reliability of biochemical results, rather than the sole method. In subsequent work, we will consider introducing more samples and other medical conditions as control conditions to improve the detection accuracy of our method presented in this paper. Meanwhile, further research is needed on the application of new feature extraction methods and classifiers in MA addiction classification.

## Conclusion and Future Work

This work proposed to detect MA abusers using time-domain, frequency-domain and wavelet features, and the highest classification accuracy rate of several classifiers was 83.85%. The classification effect of BiLSTM classifier was better than SVM. Among them, the wavelet coefficient of P300 was the main feature because the wavelet transform can decompose EEG signals of different resolutions, and the most accurate description of P300 can be obtained. Since the frequency range of P300 components is basically concentrated in the delta (0–4 Hz) band, we obtained the wavelet coefficients of 0–3.9 Hz by wavelet decomposition as features. Furthermore, the F_score was used to optimize the features set. This study can provide a judgment method for the abstinence effect of MA abusers in the future. There is currently no suitable detection method to judge whether a person has taken MA in the early stage, which is usually detected after he has reached the level of addiction (Turnip et al. 2018). Therefore, this study can provide a judgement basis for the prevention of MA addiction. In future studies, the other component of EEG signals such as N300, P200 and N200 can be analyzed jointly. Although the difference between the two groups of people regarding those components is less than that of P300, it may contain some critical factors that do not present in P300.

## Data Availability

The datasets analyzed during the current study are not publicly available, but are available from the corresponding author on reasonable request.

## Declarations

### Code Availability

The code generated during the current study are not available.
